# Effect of *In Vitro* Transcorneal Approach of Aceclofenac Eye Drops through Excised Goat, Sheep, and Buffalo Corneas

**DOI:** 10.1155/2015/432376

**Published:** 2015-01-13

**Authors:** Vivek Dave, Sarvesh Paliwal, Sachdev Yadav, Swapnil Sharma

**Affiliations:** Department of Pharmacy, Banasthali University, Banasthali 304022, India

## Abstract

The current study involves the evaluation of factors that influence the transcorneal permeation of aqueous drops of aceclofenac ophthalmic formulation through freshly excised goat, sheep, and buffalo corneas. Aceclofenac formulation with different concentrations 0.1–0.5% (w/v) and with different pH and different preservatives, was taken into account. The amount of drug permeated from different formulations was estimated using an Franz diffusion cell. A linear increase in drug permeation was observed with increase in pH (5.5 to 7.4). The apparent permeability coefficient was found to be maximum 15.01 ± 0.45 on goat cornea and maximum transport of aceclofenac was observed at physiological pH of tears (i.e., 7). The results advocate that aceclofenac 0.5% (w/v) ophthalmic solution (pH 7.0) containing BAK (0.01%) provides maximum *in vitro* ocular permeability through goat, sheep, and buffalo corneas.

## 1. Introduction

The field of ocular drug delivery is one of the interesting and challenging endeavors facing the pharmaceutical scientist. The cornea is a transparent tissue in the eye that is responsible for the refraction of incoming light and is a multilayered tissue made up of three major cell layers: the epithelium, the stroma, and the endothelium [1]. A corneal epithelium is a stratified cell membrane and its apical tight junctions between surface epithelial cells are considered to be the most prominent barrier for corneal absorption. Topical delivery into the conjunctival cul-de-sac is by far the most common route of ocular drug [2]. Absorption from this site may be corneal or noncorneal. The corneal absorption represents the major mechanism of absorption for most therapeutic entities. The cornea is a trilaminate structure consisting of three major diffusional barriers, epithelium, stroma, and endothelium [3]. Nonsteroidal anti-inflammatory drugs (NSAIDs) are recommended for treatment of various pains, inflammatory conditions of eye, osteoarthritis, and rheumatoid arthritis. It acts by blocking cyclooxygenase pathway. NSAIDs have proven to be a safe and effective alternative to corticosteroids in the topical management of ocular inflammations [4]. For treating ocular diseases, eye drops may be used but requires frequent instillation of highly concentrated solutions due to rapid precorneal loss from the eye. So, a prolonged action pharmaceutical may be recommended. Aceclofenac eye drop is not available in market so in this we use a novel approach to formulate aceclofenac eye drops to reduce eye inflammation. Currently these drugs are used topically varying widely in inhibition of intraoperative miosis, management of postoperative inflammation, treatment of seasonal allergic conjunctivitis, prevention and treatment of cystoid macular edema, and the control of pain after photo refractive keratectomy. NSAIDs have also been found to be useful in decreasing bacterial colonization of contact lenses and preventing bacterial adhesion to human corneal epithelial cells [5, 6].

Rabbit cornea has been preferred in the majority of the permeation studies which is now been restricted by most of Animal Ethical Committees across the globe. Keeping this view in mind, three different corneas of goat, sheep, and buffalo were selected for this study. Corneas were procured from local butcher's shop at Banasthali, Newai, Rajasthan. The current study focuses on different factors like pH, nature and amount of preservatives, that influences rate of permeation (*in vitro*) of an aceclofenac formulation through different freshly excised corneas of goat, sheep, and buffalo.

## 2. Material and Method

### 2.1. Materials

Aceclofenac was obtained from (Lupin Research Park, Pune). All preservatives and chemicals purchased were of analytical reagent grade, CDH (New Delhi, India). Fresh and healthy whole eyeballs of goat, sheep, and buffalo were acquired from butcher's shop (Banasthali, Newai, Rajasthan, India) preferably 30 minutes after animal slaughtering.

### 2.2. Corneal Preparation

**Figure 1:**
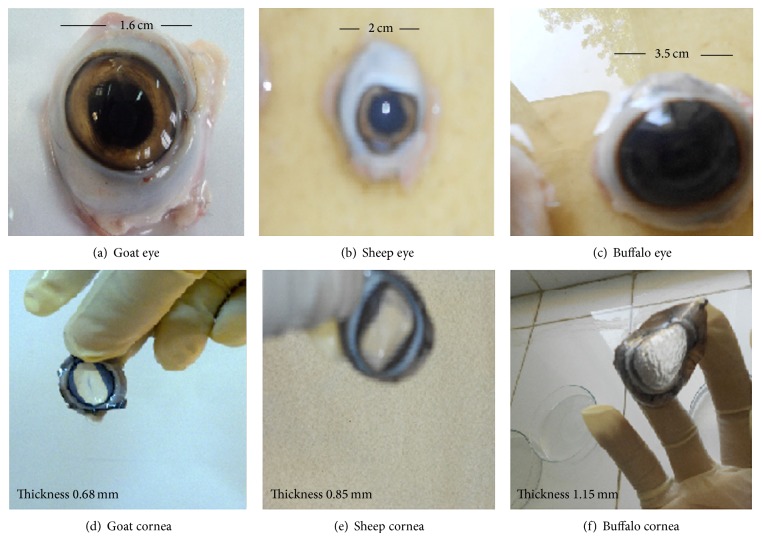
Optical observation: (a) goat eye, (b) sheep eye, (c) buffalo eye, (d) goat cornea, (e) sheep cornea, and (f) buffalo cornea.

Freshly excised whole eyeballs of goat, sheep, and buffalo were procured from local butcher's shop to laboratory in cold (4°C) saline within 1 h of slaughtering. The corneas were carefully dissected along with 2–4 mm of surrounding sclera tissue from the eyeball and washed with cold saline so as to remove any adhering pigments as shown in Figure 1. The washed cornea was preserved in freshly prepared balance base buffer (pH 7.4) with % w/v composition of NaCl—0.57 g, NaHCO_3_—0.361 g, KCl—0.04 g, K_2_HPO_4_—0.023 g, MgSO_4_—0.007 g,and CaCl_2_—0.08 g in glass distilled water and bubbled with O_2_ to keep the cornea in viable state.

### 2.3. Permeation Experiment

Fresh corneas obtained by the above procedure were mounted on the modified Franz diffusion apparatus by sandwiching the scleral tissues between the clamped donor and the receiver chamber. Care was taken to maintain the convex surface shape of the cornea by suitable design of the clamp, receiver, and donor chamber edge and also to ensure that the epithelial surface of the cornea is towards the donor side. Balance base buffer (composition same as given in previous section) was filled in receiver chamber after expelling all the air bubbles by inverting the diffusion cell and then allowing the bubbles to travel through the sampling port. The receiver fluid was maintained at 37 ± 1°C with the help of circulating warm water and kept under stirring using a Teflon coated magnetic bead. An aliquot (1 mL) of test sample containing different concentrations of aceclofenac 0.1, 0.2, 0.3, 0.4, and 0.5% (w/v) was placed on the epithelial surface of each cornea in the donor chamber, respectively, and covered with glass slip using silicone grease to prevent evaporation. In the entire experiment the permeation was continued for 120 min at predetermined time points of 30, 60, 90, and 120 min, and a 1 mL sample was withdrawn through the sampling port, suitably diluted with 0.1 N HCL and analyzed by spectrophotometer method as described by Malhotra and Majumdar [6]. The concentration of permeated drug at the defined time intervals was determined using standard curve:

(1) Permeation%=Amount  of  drug  permeated  in  receptorInitial  amount  of  drug  in  donor×100.

After the completion of the experiment, all the corneas were weighed and reweighed after overnight drying at 90°C. Corneal hydration was estimated from the difference in weights of hydrated and dehydrated of cornea. Moreover aceclofenac ophthalmic aqueous solution was allowed to pass through corneas of goat, buffalo, and sheep for different time intervals for the determination of permeation characteristics.

### 2.4. Apparent Permeability Coefficient

Different solution of different concentration (0.1% to 0.5%.) of drug aceclofenac was prepared in 100 mL of isotonic phosphate buffer of pH 7.0 using different preservative containing either benzalkonium chloride (BAK 0.01% w/v), or phenyl mercuric nitrate (PMN 0.001% w/v), or benzyl alcohol (BA 0.5% v/v). All the prepared solutions were filtered, packed, and sealed in glass vials. Finally all the containers were sterilized by autoclave at 121°C for 15 min. The apparent permeability coefficient was determined using different corneas compared. Apparent permeability coefficient was also calculated using the following equation:

(2) $$\begin{array}{c} P_{\text{app}}=\frac{\Delta Q}{\Delta t}\cdot \frac{1}{A\cdot C_{0}\cdot 60}, \end{array}$$

where Δ*t* (*μ*g/min) is the flux across the corneal tissue.

*A* is the area of diffusion (cm^2^), *C*_0_ is the initial concentration of drug in donor compartment, and 60 is taken as the factor to convert minute into second. The flux across the cornea was obtained from the slope of the regression line obtained from the linear part of the curve between the amount permeated (*Q*) versus time (*t*) plot.

### 2.5. Different Formulation of Aceclofenac Ophthalmic Solution at pH 7.0

Solutions of 0.1, 0.2, 0.3, 0.4, and 0.5% (w/v) concentrations of aceclofenac were prepared by dissolving specific amount of aceclofenac in adequate isotonic phosphate buffer and diluted up to 100 mL of distilled water. 0.1 N NaOH or 0.1 N HC l was used to adjust pH at 7.0. All the prepared solutions were filtered, packed, and sealed in glass vials. Finally all the containers were sterilized by autoclave at 121°C for 15 min.

### 2.6. Formulation of Aceclofenac Ophthalmic Solutions 0.5% w/v, pH 7.0 Containing Preservative

The drug aceclofenac (0.5 g) was dissolved in 100 mL of isotonic phosphate buffer, pH 7.0 containing either benzalkonium chloride (BAK 0.01% w/v), or phenyl mercuric nitrate (PMN 0.001% w/v), or benzyl alcohol (BA 0.5% v/v) and the final volume of each solution was made up to 100 mL with distilled water. All the prepared solutions were filtered, packed, and sealed in glass vials. Finally all the containers were sterilized by autoclave at 121°C for 15 min.The apparent permeability coefficient was found to be more in goat cornea compared with sheep and buffalo corneas for all concentration of aceclofenac eye drops 0.1% to 0.5%.

### 2.7. Determination of Surface Tension

Presence of surfactant in formulation may emulsify the epithelial layer of cornea and assist in more rapid partitioning of the drug in the same layer. Surface tension of each aceclofenac ophthalmic solution (0.5% w/v, pH 7.0) was determined using stalagmometer, to establish a correlation between surface tension of formulation and corneal penetration.

### 2.8. Measurement of Partition Coefficient (log⁡*p*)

10 mg of aceclofenac drug was added in 50 mL of *n*-octanol (presaturated with water) and then 50 mL of distilled water (pre saturated with *n*-octanol) was added. The process was continued in mechanical shaker for 24 hours. After 24 hour both phases were separated. Absorbance was taken of both the phases and calculated the concentration in each phase, that is, log⁡*P* = [aceclofenac]oct/[aceclofenac] H_2_O. The concentration of the aceclofenac base dissolved in *n*-octanol was obtained by extrapolation from a calibration curve (0–20 *μ*g/mL) of the aceclofenac base in *n*-octanol at 275 nm (*λ* max) [7–9].

### 2.9. Isotonicity Evaluation

The tonicity of the eye drops was checked by mixing the eye drops with citrated blood and observed under the microscope (45x) for the effect on RBC for cremation or swelling and bursting.

## 3. Results and Discussion

The cornea of the eye has three distinct layers (from inner to outer), that is, endothelium (less lipophilic than epithelium), stroma (hydrophilic), and epithelium (lipophilic). The corneal tissues were assumed to be effectively represented by plane sheet barriers of physiological thickness. The tear film was assumed to be absolutely mixed and the stroma completely stagnant. Due to inadequate knowledge of the hydrodynamics of the aqueous humour, both stagnant and perfectly mixed extremes were studied. The equilibrium that can exist between the ionic and nonionic forms of a drug was found to be an important step in the mechanism of transcorneal permeation [8, 9].

Permeation statistics of aceclofenac from ophthalmic solutions of increasing concentrations through three different excised goat, sheep, and buffalo corneas are represented in Table 1. The data reveals that the drug permeability at particular pH linearly increases with the concentration of aceclofenac from 0.1 to 0.5%. It is worth mentioning that though an increase in permeation was observed with incremental concentration of drug, at the same time marked reduction in percentage permeation also occurred. The apparent permeability coefficient was found to be more in goat cornea compared with sheep and buffalo cornea for all concentration of aceclofenac eye drops 0.1% to 0.5%. The effects of different preservatives on permeation of aceclofenac ophthalmic aqueous solution through excised goat, sheep, and buffalo corneas were also evaluated and the results are shown in Table 2.* In vitro* relative permeation of aceclofenac from control and optimized formulation through excised goat, sheep, and buffalo corneas data indicates that maximum release was achieved across goat cornea (84%) and minimum with buffalo cornea (34%). In contrast to this when compared with control formulation containing no preservative percentage permeation showed 56% on goat, 24% on sheep, and 22% on buffalo. The apparent permeability coefficient was found to be more in goat cornea compared with sheep and buffalo cornea for all preservative containing aceclofenac eye drops. It is evident from the result that use of BAK, a cationic surfactant and methyl paraben and propylparaben, showed a significant augment in permeation was observed. Likewise, formulation containing thiomersal demonstrated minor increase in permeation, whereas those with phenyl mercuric acetate did not have any consequence on permeation. The mutual presence of methyl paraben and propylparaben or BAK in the formulation resulted in utmost permeation of the drug through all the three corneas. Nonetheless, Sieg and Robinson [10] and Madhu et al. [11] also reported that corneal epithelium acts as a reservoir for drug accumulation and provides continuous delivery of drug to aqueous humor in context to permeation studies of ketorolac and pilocarpine. The increased permeation with formulation containing BAK appears to be caused by emulsification of epithelial layer of cornea and enhancement of lipid solubility of aceclofenac. The corneal hydration of aceclofenac eye drops through goat, sheep, and buffalo corneas was found to be from 79 ± 0.342% to 80.9 ± 0.486%, respectively. An attempt was also made to check permeation of optimized formulation containing BAK and control formulation (without benzalkonium chloride) through paired corneas of goat, sheep, and buffalo.

To minimize biological variation paired corneal study was carried out. In paired corneal study, both eyes from an animal were taken to get cornea and received different treatment. One of the corneas was received with optimized formulation containing BAK whereas the other cornea was treated with control formulation containing additive. The augmented permeation of aceclofenac was observed with formulation containing BAK through all the distinct corneas when compared with the control formulation in Table 2. Similar result was reported to increase the permeation of moxifloxacin through excised goat, sheep, and buffalo corneas [12–14]. Surface tension was observed between 53.1 and 69.4 dyne/cm. The formulation containing BAK as preservative was found to be most significant amongst all. The *n*-octanol/water partition coefficient (log⁡*p*) of aceclofenac drug was found to be 1.86 ± 0.75.

**Figure 2:**
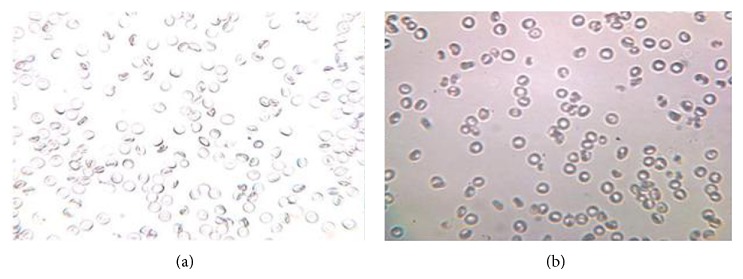
(a) Red blood cell and (b) red blood cell with optimized formulation aceclofenac eye drops containing HPMC.

The tonicity of the eye drops was checked by mixing the eye drops with citrated blood and observed under the microscope (45x) for the effect on RBC for cremation or swelling and bursting. Isotonicity is an important characteristic of the ophthalmic preparation. Isotonicity has to be maintained to prevent tissue damage or irritation of eye. Since our optimized aceclofenac eye drops formulation containing hydroxypropylemethylcellulose exhibited good and prolonged release characteristics, it was subjected to isotonicity testing. Formulations were mixed with few drops of blood and observed under microscope at 45x magnification and compared with RBC alone. Isotonicity testing of aceclofenac eye drops having BAK exhibited no change in the shape of blood cells (bulging or shrinkage), which reveals the isotonic nature of the formulation as showed in Figure 2. The thickness of the corneas was also observed as shown in Table 3 and Figure 1. The thicknesses of goat, sheep, and buffalo cornea were found to be 0.68 ± 0.0003 mm, 0.86 ± 0.0003 mm, and 1.12 ± 0.0006 mm, respectively. In fact, all the marketed eye drops contained BAK, which is known to increase the corneal permeation of the drug by disruption of the corneal epithelium [15–18].

### 3.1. Statistical Analysis

One-way ANOVA followed by Dunnett's test was applied. Paired *t*-test was preferred used for with paired corneal studies. *P* ≤ 0.05 was considered as criterion for significance.

## 4. Conclusion

The present studies demonstrated the influence of different concentration aceclofenac aqueous drops on its permeation rate (*in vitro*) through three different mammalian corneas. The maximum transport of aceclofenac was observed at physiological pH of tears (i.e., 7). Aceclofenac 0.5% w/v aqueous drops (pH 7.0), containing BAK (0.01% w/v), showed maximum* in vitro* ocular accessibility through goat, sheep, and buffalo corneas.

## Figures and Tables

**Table 1:** *In vitro* permeation studies of different concentrations of aceclofenac in aqueous solution through excised goat, sheep, and buffalo corneas.

Conc. % (w/v)	Amount permeated (mg) (120 minutes)			Permeation (%) (120 minutes)			Corneal hydration (%)			Papp cm/sec ∗ 10^6^		
	Goat	Sheep	Buffalo	Goat	Sheep	Buffalo	Goat	Sheep	Buffalo	Goat	Sheep	Buffalo
0.1	1.006 ± 0.005	0.944 ± 0.002	0.789 ± 0.004	98	94.4	78.90	80.2 ± 0.582	80.7 ± 0.001	80.2 ± 0.68	13.81 ± 0.2	12.06 ± 0.02	10.15 ± 0.1
0.2	1.8 ± 0.0012	1.6 ± 0.003	1.62 ± 0.001	90	80	81	80.5 ± 0.021	80.5 ± 0.245	79 ± 0.366	11.55 ± 0.1	10.27 ± 0.1	10.3 ± 0.8
0.3	2.05 ± 0.004	1.96 ± 0.001	1.87 ± 0.003	68.33	65.5	62.4	80.1 ± 0.187	80.9 ± 0.548	80.4 ± 0.366	9.15 ± 0.02	8.78 ± 0.4	8.42 ± 0.6
0.4	2.43 ± 0.006	2.36 ± 0.002	2.24 ± 0.001	60.75	59	56.0	80.4 ± 0.456	80.1 ± 0.216	77 ± 0.325	7.89 ± 0.8	8.41 ± 0.3	7.24 ± 0.2
0.5	2.73 ± 0.001	2.64 ± 0.005	2.58 ± 0.008	54.6	52.8	51.6	79.2 ± 0.213	81.2 ± 0.658	79.3 ± 0.39	7.24 ± 0.12	7.00 ± 0.2	6.8 ± 0.4

Values are mean ± SE of three corneas in each group. Statistically significant (*P* < 0.05) determined by one-way ANOVA followed by Dunnett's test.

Note: All the experiments were carried out in triplicates.

**Table 2:** * In vitro* permeation studies of 0.5% aqueous solution of aceclofenac from with different preservatives through excised goat, sheep, and buffalo cornea.

Preservative	Amount permeated (mg) (120 minutes)			Permeation (%) (120 minutes)			Corneal hydration (%)			Papp cm/sec ∗ 10^6^			Surface tension (dyne/cm)
	Goat	Sheep	Buffalo	Goat	Sheep	Buffalo	Goat	Sheep	Buffalo	Goat	Sheep	Buffalo	
Control	2.8 ± 0.002	1.2 ± 0.005	1.1 ± 0.004	56	24	22	79.1 ± 0.411	80.4 ± 0.001	79.2 ± 0.55	9.998 ± 0.86	3.36 ± 0.12	2.99 ± 0.36	69.4
BAC	4.4 ± 0.004	2.8 ± 0.008	1.7 ± 0.005	84	76	34	80.5 ± 0.145	78.5 ± 0.548	80.4 ± 0.312	15.01 ± 0.45	9.60 ± 0.03	4.91 ± 0.21	53.1
MP-PP	4.1 ± 0.003	3.6 ± 0.004	1.4 ± 0.003	82	64	28	80.2 ± 0.021	80.1 ± 0.245	79 ± 0.342	15.09 ± 0.40	8.71 ± 0.05	5.09 ± 0.12	54.7
BA	3.2 ± 0.005	2.4 ± 0.004	1.5 ± 0.009	63	50	30	80.9 ± 0.486	80.1 ± 0.116	79 ± 0.325	8.65 ± 0.21	7.01 ± 0.06	5.26 ± 0.32	64.1
THM	1.6 ± 0.009	1.3 ± 0.007	1.1 ± 0.001	32	26	22	79.2 ± 0.125	80.2 ± 0.658	80.3 ± 0.314	5.23 ± 0.45	3.48 ± 0.12	2.99 ± 0.68	60.8
PMA	1.7 ± 0.001	1.1 ± 0.006	1.0 ± 0.001	34	22	20	80.27 ± 0.129	80.2 ± 0.448	80.69 ± 0.314	6.454 ± 0.65	2.99 ± 0.15	2.55 ± 0.45	65.1

BAK indicates benzalkonium chloride; MP-PP, combination of methyl paraben and propylparaben; BA, benzyl alcohol; THM, thiomersal; PMA, phenyl mercuric acetate; values are mean ± SE of three corneas in each group. Statistically significant (*P* < 0.05) determined by one-way ANOVA followed by Dunnett's test.

Note: All the experiments were carried out in triplicates.

**Table 3:** *In vitro* relative permeation characteristics of aceclofenac from control and optimized formulation through excised goat, sheep, and buffalo corneas.

Animal	Thickness of cornea mm	Control formulation			Optimized formulation BAC benzalkonium chloride		
		Amount permeated (mg) (120 minutes)	Permeation (%) (120 minutes)	Papp cm/sec ∗ 10^6^	Amount permeated (mg) (120 minutes)	Permeation (%) (120 minutes)	Papp cm/sec ∗ 10^6^
Goat	0.68 ± 0.0003	2.8 ± 0.002	56	9.998 ± 0.86	4.4 ± 0.004	84	15.01 ± 0.45
Sheep	0.86 ± 0.0003	1.2 ± 0.009	24	3.36 ± 0.12	2.8 ± 0.008	76	9.60 ± 0.03
Buffalo	1.12 ± 0.0006	1.1 ± 0.004	22	2.99 ± 0.36	1.7 ± 0.005	34	4.91 ± 0.21

Values are mean ± SE of three corneas in each group. Statistically significant (*P* < 0.05) determined by one-way ANOVA followed by Dunnett's test.

Note: All the experiments were carried out in triplicates.
